# Two-step complete polarization logic Bell-state analysis

**DOI:** 10.1038/srep13453

**Published:** 2015-08-26

**Authors:** Yu-Bo Sheng, Lan Zhou

**Affiliations:** 1 Institute of Signal Processing Transmission, Nanjing University of Posts and Telecommunications, Nanjing, 210003, China; 2 Key Lab of Broadband Wireless Communication and Sensor Network Technology, Nanjing University of Posts and Telecommunications, Ministry of Education, Nanjing, 210003, China; 3 College of Mathematics & Physics, Nanjing University of Posts and Telecommunications, Nanjing, 210003, China

## Abstract

The Bell state plays a significant role in the fundamental tests of quantum mechanics, such as the nonlocality of the quantum world. The Bell-state analysis is of vice importance in quantum communication. Existing Bell-state analysis protocols usually focus on the Bell-state encoding in the physical qubit directly. In this paper, we will describe an alternative approach to realize the near complete logic Bell-state analysis for the polarized concatenated Greenberger-Horne-Zeilinger (C-GHZ) state with two logic qubits. We show that the logic Bell-state can be distinguished in two steps with the help of the parity-check measurement (PCM) constructed by the cross-Kerr nonlinearity. This approach can be also used to distinguish arbitrary C-GHZ state with *N* logic qubits. As both the recent theoretical and experiment work showed that the C-GHZ state has its robust feature in practical noisy environment, this protocol may be useful in future long-distance quantum communication based on the logic-qubit entanglement.

Bell-state analysis (BSA) is of vice importance in quantum communication. Quantum teleportation^1^, quantum key distribution^2^, and quantum secure direct communication^3^,^4^ all need the BSA. Especially, in long-distance quantum communication, in order to resist the environmental noise, they should exploit the entanglement swapping instead of distributing the photon directly to extend the length of the entanglement, which is called the quantum repeaters^5^. The key element of the quantum repeaters is still the BSA.

Usually, in an optical system, there are three different approaches to realize the BSA. The first approach requires the linear optical elements^6^,^7^,^8^. However, one cannot perform the complete BSA with only linear optics, for the optimal success probability is only 50%^6^,^7^. The second approach still requires the linear optical elements but resorts to the hyperentanglement^9^,^10^,^11^,^12^. For example, Walborn *et al.* described a feasible and interesting hyperentanglement-assisted BSA protocol^9^. In their protocol, the hyperentangled state is prepared in both polarization and momentum degrees of freedom. With the help of momentum-entangled Bell state, they can completely distinguish four polarized Bell states. Their protocol can also distinguish four momentum-entangled Bell states, with the help of polarization Bell state. In 2008, the group of Kwiat beat the channel capacity limit for linear photonic superdense coding^12^. They can completely distinguish four polarized Bell states with orbital-angular momentum entanglement. In essence, this approach works in a large Hilbert space in two degrees of freedom. The third approach works in a nonlinear optical system^13^,^14^,^15^,^16^. For instance, with the help of the cross-Kerr nonlinearity, they can perform the near complete parity-check measurement (PCM)^13^,^17^. The PCM can distinguish the even parity states 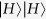 and 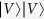 from the odd parity states 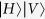 and 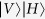 near deterministically. Here 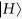 is the horizonal polarized photon and 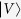 is the vertical polarized photon, respectively. The complete polarization BSA can be well performed in two steps. The first step is to distinguish 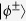 from 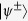. The second step is to distinguish 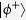 from 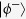, and 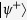 from 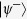, respectively. Here 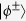 and 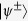 are four polarized Bell states of the form


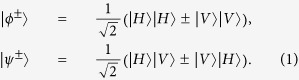


On the other hand, it is known that the decoherence is one of the main obstacles in long-distance quantum communication. In the past decades, people developed serval approaches to resist the decoherence. They presented the quantum repeaters^5^ and nonlinear photon amplification^18^,^19^,^20^ to resist the photon loss during the entanglement distribution. They also proposed the entanglement purification^21^,^22^,^23^,^24^,^25^,^26^,^27^,^28^,^29^,^30^ and concentration^31^,^32^,^33^,^34^,^35^ to improve the quality of the degraded entanglement. In current quantum communication protocols, they usually encode the quantum qubit in the physical qubit directly. In 2011, Fröwis and Dür developed a class of quantum entanglement, which encodes many physical qubits in a logic qubit^36^. Such logic-qubit entanglement has the similar feature as the Greenberger-Horne-Zeiglinger (GHZ) state, but is more robust than the normal GHZ state in a noisy environment. In 2012, Munro *et al.* developed a new approach of quantum communication based on logic qubits^37^. The logic-qubit entanglement, which is also called the concatenated GHZ (C-GHZ) state can be described as^38^,^39^,^40^,^41^,^42^


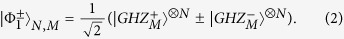


Here *N* is the number of logic qubit and *M* is the number of the physical qubit in each logic qubit. 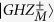 are the *M*-photon polarized GHZ states as


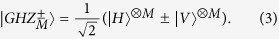


In 2014, Lu *et al.* realized the first experiment of C-GHZ state with *N* = 2 and *M* = 3 in a linear optical system^42^. By observing the dynamics of distillability of the C-GHZ evolving under a collective noisy environment, they showed that the C-GHZ state can tolerate more noise than the GHZ state.

As the logic-qubit entangled state is more robust than the entanglement encoded in the physical qubit directly, it is possible to perform the quantum communication based on logic-qubit entanglement. Due to the importance of BSA in quantum communication, it is interesting to discuss the BSA encoded in logic qubit. One line of the research for logic Bell-state analysis (LBSA) in based on linear optics. For example, recently, Lee *et al.* described the LBSA for another type of logic qubit. Their protocol is based on the linear optics and does not require photon-number-resolving measurements, which is feasible in current experimental condition^43^. Another line of research exploits the cross-Kerr nonlinearity. For example, we will describe an approach to realize the near complete LBSA based on logic-qubit entanglement. We also show that this approach can be used to perform the arbitrary C-GHZ state analysis (Supplementary Materials). Such LBSA will provide us some interesting application in future quantum information processing, such as the quantum teleportation and entanglement swapping for an arbitrary logic qubit. In this way, we can set up the long-distance quantum entanglement channel based on the logic-qubit entanglement.

## Results

In this section, we will start to explain our LBSA. A logic Bell state can be regarded as the special state of the C-GHZ state with *N* = *M* = 2. A logic Bell state contains two logic qubits. Each logic qubit is encoded in a polarized Bell state. The four logic Bell states can be described as


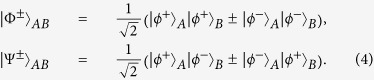


**Figure 1:**
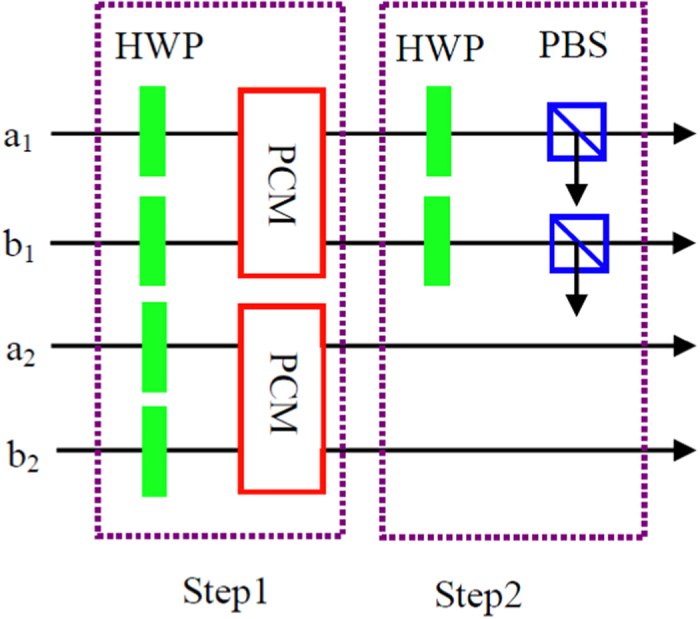
A schematic drawing of our LBSA. PCM represents the parity-check measurement gate described in Method section.

From Fig. 1, the two photons in logic qubit A are in the spatial modes a_1_ and a_2_, respectively. The two photons in logic qubit B are in the spatial modes b_1_ and b_2_, respectively. We first let four photons pass through the half wave plates (HWPs), which will make 
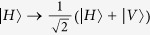
 and 
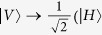
 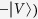. The HWPs act as the role of Hadamard operation. The four HWPs will transform the states in Eq. (4) to


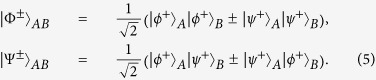


After passing through the HWPs, the state 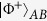 can be described as


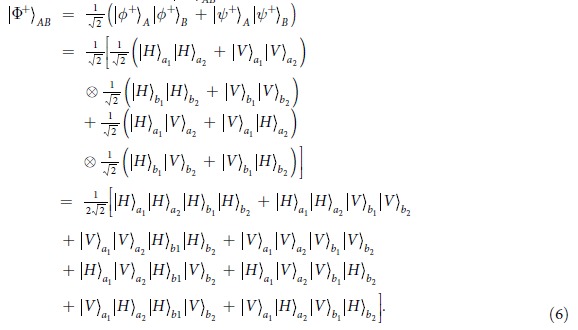


In the first step, we let the four photons in spatial modes *a*_1_ and *b*_1_, *a*_2_ and *b*_2_ pass through the two PCM gates, respectively. The PCM gate is detailed in the Method Section. Briefly speaking, in each PCM gate, the two photons combined with the coherent state 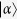 will couple with the cross-Kerr material. The PCM gate can distinguish the even parity states 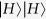 and 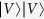 from the odd parity states 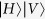 and 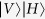, by measuring the phase shift of the coherent state. If the coherent state picks up no phase shift, we will get the even parity state. On the other hand, if the coherent state picks up 2*θ* phase shift, we will get the odd parity state. During the whole measurement, we do not measure the two photons directly. Such function is also described in Ref. 13. Interestingly, if the initial state is 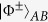, after performing the measurements, the results of the two PCMs are the same. If the PCM results are both even, 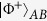 will become


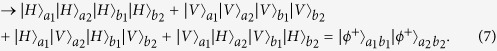


While if the PCM results are both odd, 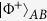 will become


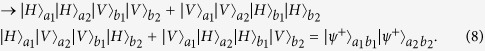


On the other hand, if the initial state is 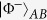, we can obtain the same results as 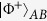. In detail, if the PCM results are even, 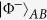 will collapse to


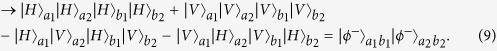


while if the measurement results are both odd, 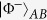 will become
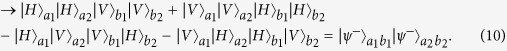
If the initial state is 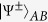, after performing the PCM operations, the measurement results of the two PCM gates are different. If the PCM result in spatial modes a_1_ and b_1_ is even, the PCM result in spatial modes a_2_ and b_2_ must be odd. While if the PCM reslut in spatial modes a_1_ and b_1_ is odd, the PCM result in spatial modes a_2_ and b_2_ must be even. In the first case, the 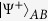 will collapse to 
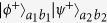
 and 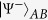 will collapse to 
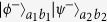
. In the second case, 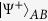 will collapse to 
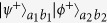
 and 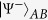 will collapse to 
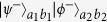
.

From above description, according to the PCM results, the four logic Bell states can be divided into two groups. If the PCM results are the same, they are 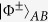. If the PCM results are different, they are 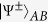. The second step is to distinguish 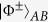 or 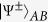 in each group. We take 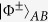 for example. From Eqs (7 and 9), if the initial state is 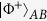, the state in a_1_b_1_ must be 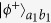 Otherwise, if the initial state is 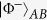, the state in a_1_b_1_ must be 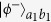. Therefore, the second step only needs to distinguish the states 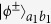. After two photons passing through the two HWPs, state 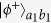 will not change, while 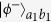 will become 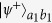. Finally, we let two photons pass through two polarization beam splitters (PBSs), respectively. The PBS can transmit 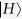 polarized photon and reflect 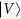 polarized photon, respectively. 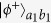 will make two photons both transmit or reflect from two PBSs, but 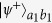 will make one photon transmit from the PBS, and the other reflect from the PBS, respectively. According to the output modes of the two photons, we can easily distinguish 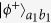 from 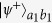. If the initial states are 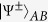, they can be distinguished in the same way. In this way, the four logic Bell states can be completely distinguished.

## Discussion

So far, we have fully described our LBSA. This approach can be extended to perform the C-GHZ state analysis (see Supplementary Materials). In the LBSA, two PCM gates are required. In the first step, two PCM operations on the *a*_1_*b*_1_ and *a*_2_*b*_2_ spatial modes are both performed. According to the measurement results, we can distinguish the states 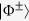 from 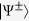. If the measurement results are the same, the original states must be 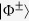. Otherwise, the original states must be 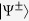. In the second step, we only need to distinguish the conventional polarized Bell state 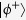 from 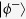. It can be well distinguished by two PBSs. In this way, the four logic Bell states can be completely distinguished. In above explanation, we let the logic qubits are 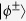. If the logic qubits are arbitrary polarized GHZ states 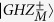, this LBSA can also be well performed (see Supplementary Materials).

It is known that BSA plays an important role in quantum communication. If the LBSA can be well performed, it may provide us an additional application in quantum communication. For example, we can teleportate an arbitrary logic qubit 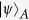. Suppose the logic qubit 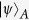 is


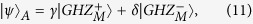


with 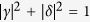. The logic qubits *B* and *C* are the logic-qubit entanglement with
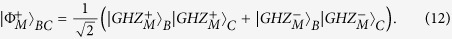
The logic qubit *A* combined with the logic-qubit entanglement *B* and *C* can be written as


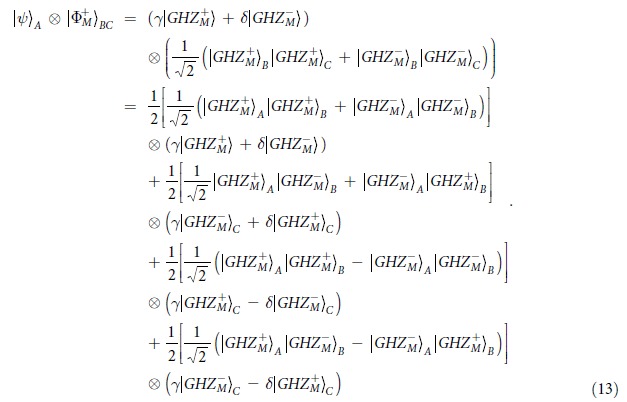


From Eq. (13), if we can well perform the LBSA on logic qubit *A* and *B*, we can teleportate the logic qubit *A* to *C*. Briefly speaking, if the LBSA result is 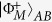, logic qubit *C* has the same form of the original qubit *A*, which is defined as 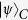. If the LBSA result is 
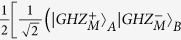
 
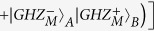
, the logic qubit *C* is 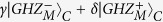. It can be transformed to 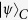 by performing a phase flip operation on one of the photons. If the LBSA result is 
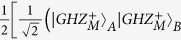
 
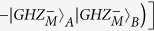
, the logic qubit *C* is 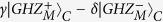, one can perform bit-flip operation on all the photons to transform it to 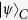. Finally, if the LBSA result is 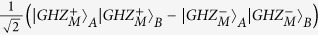, one can also transform the state 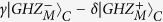 to 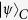. In this way, the logic qubit teleportation can be completely performed.

We can also perform the entanglement swapping with logic-qubit entanglement. Quantum repeaters based on logic-qubit entanglement will provide the double protection from the environmental noise. Suppose that the logic-qubit entanglement *A* and *B*, and *C* and *D* are both 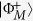. The whole system can be described as


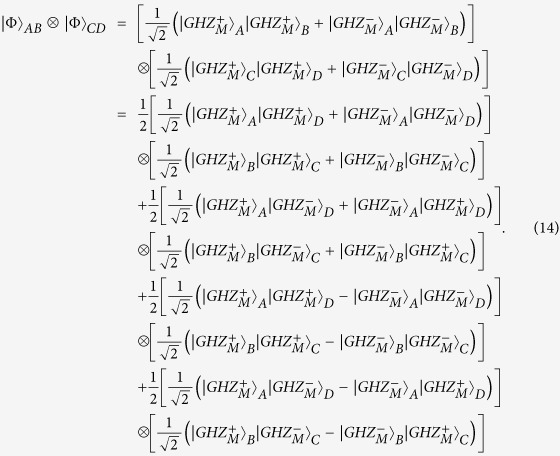


From Eq. (14), if we perform the LBSA on logic qubit *B* and *C*, we can connect the entanglement between the logic qubit *A* and *D*.

In our protocol, the key element to realize the LBSA is the PCM gate, which is constructed by the cross-Kerr nonlinearity. As pointed out by Refs 17, 44, the error probability of the PCM gate can be described as 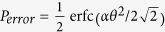, where *α* is the parameter of the coherent state 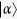 and *θ* is the phase shift of the coherent state. *P*_*error*_ can be explained as follows. The parity of the measured state is even, but the PCM shows odd, with the probability *P*_*error*_. On the other hand, the parity of measured state is odd, but the PCM shows even, with the same probability *P*_*error*_. In LBSA, if the logic qubits are 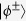, we can calculate the fidelity of LBSA as


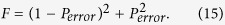


Here the fidelity is denoted as the probability to perform the correct LBSA. As shown in Fig. 1, we are required to perform PCM twice. Suppose that the initial state are one of the states 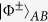. After performing the PCM operations, the initial state collapses to the state in Eq. (7). During such precess, if both PCM results are even, with the right probability of (1 − *P*_*error*_)^2^, we will judge the right state in Eq. (7). However, if both PCM results are odd, with the error probability of 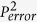, we will judge the error state in Eq. (8). Actually, the collapsed state is still the state in Eq. (7). Interestingly, both states in Eq. (7) and 8 mean that the original state are 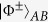. It reveals that even if both PCM results are wrong, it does not affect the discrimination. Certainly, if one of PCM is right and the other is wrong with the probability of 2*P*_*error*_(1 − *P*_*error*_), which shows that the PCM results are different, it will mislead us that the original state is 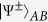. In this way, it contributes the error discrimination.

Interestingly, in the C-GHZ state analysis, the discrimination number of the states is 2^*N*^, which is decided by the logic qubit number *N*. The physical qubit number *M* does not affect the discrimination number of the state. Using single-qubit rotation and single-qubit measurement, we can transform 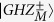 to 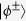 by measuring *M* − 2 photons in each logic qubit. If we only consider that the error coming from the PCM, the number *M* does not affect the total fidelity. However, the number of logic qubit *N* will decrease the fidelity. It can be written as


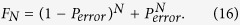


**Figure 2:**
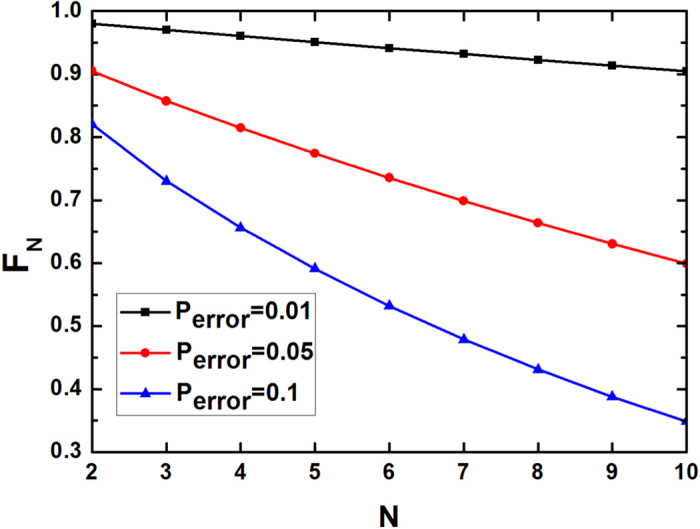
Schematic of the fidelity of the C-GHZ state analysis altered with the logic qubit number *N*. The *P*_*error*_ is 0.01, 0.05, and 0.1, respectively.

As pointed out in Refs 13, 44, highly accurate discrimination is possible with weak cross-Kerr nonlinearities. In an NV-diamond system, such error probability can reach *P*_*error*_ = 0.01. In Fig. 2, we calculated the fidelity *F*_*N*_ altered with the logic qubit number *N*. We let *P*_*error*_ be 0.01, 0.05 and 0.1, respectively. From Fig. 2, if *P*_*error*_ = 0.01, we can obtain *F*_*N*_ ∼ 0.9 with *N* = 10. Another imperfection comes from the detection efficiency. The detection efficiency means that the single photon enters the single-photon detector, but the single-photon does not register it with the probability of *η*. Therefore, the success probability of registering a single photon is 1 − *η*. From Fig. 1, the detection efficiency will decrease the total success probability. For the LBSA with *N* = *M* = 2, the success probability is *P*_2,2_ = (1 − *η*)^2^. We can also obtain *P*_*N*,2_ = (1 − *η*)^*N*^ with *M* = 2. If *M* > 2, we first convert the states 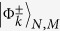 to 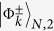 by measuring *M* − 2 photons in each logic qubit. Here *k* = 1, 2, …, 2^*N* − 1^. Such success probability is [(1 − *η*)^*M* − 2^]^*N*^. Therefore, the total success probability is *P*_*N*,*m*_ = (1 − *η*)^(*M* − 2)*N*^. It shown that if *M* > 2, it will greatly decrease the total success probability. For example, if *η* = 0.1, *P*_3,2_ ≈ 0.73, while *P*_3,3_ ≈ 0.387.

On the other hand, though the robustness of the C-GHZ state was discussed both by theory and experiment, there is still a controversial topic. For example, Chaves *et al.* did not acknowledge that the simpler GHZ encodings is robust against simpler dephasing noise^45^,^46^, which requires us to perform further investigation. Moreover, though there are many theoretical works for quantum information processing based on cross-Kerr nonlinearity, the cross-Kerr nonlinearity is also a controversial topic^47^. The debate over the usefulness of photonic quantum information processing based on the cross-Kerr nonlinearity is that the phase shift is too small to be measured in a single photon level. It is a typical dimensionless magnitude of *θ* ∼ 10^−18^. Fortunately, the theoretical work showed that with electromagnetically induced transparencies (EIT), whispering-gallery microresonators, optical fibers, or cavity QED systems, nonlinearities of magnitude *θ* can reach *θ* ∼ 10^−2^ 13,44. Moreover, some recent researches also showed that it is possible to obtain the observable value of the Kerr phase shift^48^,^49^,^50^.

In conclusion, we have described a two-step approach to realize the near complete logic Bell-state and arbitrary C-GHZ state analysis. In our protocol, we exploit the cross-Kerr nonlinearity to construct the PCM gate. The whole task can be divided into two steps. In the first step, after performing the PCM operations, the four states can be divided in two groups. The first group is 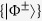 and the second group is 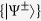. In the second step, the states 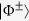 and 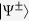 in each group can also be discriminated by PCM operation. Our protocol can be extended to distinguish the arbitrary C-GHZ state. It can also be divided into two steps. In the first step, all the 2^*N*^ C-GHZ states can be divided into 2^*N* − 1^ groups, according to the different PCM results in both left and right sides. In each group, the two states can also be completely distinguished in the second step. We also discussed the potential application of this LBSA, such as teleportating a logic qubit and performing the logic-qubit entanglement swapping. This LBSA may provide an alternative approach to perform the other quantum communication tasks, such as quantum key distribution, quantum secure direct communication, quantum state sharing, and so on.

## Methods

Cross-Kerr nonlinearity provides us a powerful tool to construct the PCM gate, which has been widely used in quantum information processing. There are many researches based on the cross-Kerr nonlinearity, including the construction of the controlled-not (CNOT) gate^17^, performing the quantum communication^51^, quantum computation^52^, and the BSA^13^, realizing the entanglement purification^22^ and concentration^33^, and so on^44^,^53^,^54^,^55^,^56^.

**Figure 3:**
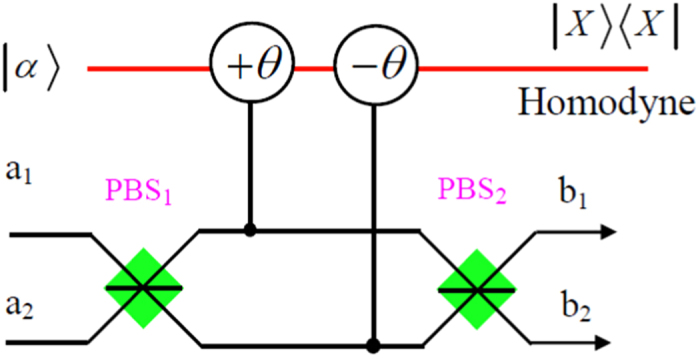
A schematic drawing of our PCM gate. It can distinguish the even parity states 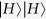 and 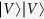 from the odd parity states 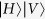 and 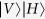. PBS represents the polarization beam splitters which can transmit the 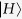 photon and reflect the 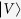 photon. The similar PCM gate is also shown Ref. 17.

As shown in Fig. 3, the Hamiltonian of a cross-Kerr nonlinear medium can be written as 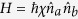. The 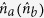 is the number operator for mode *a*(b)^17^. The 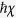 is the coupling strength of the nonlinearity. It is decided by the cross-Kerr material. If we consider a two-photon state 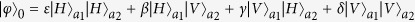. Here 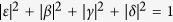 and *a*_1_(*a*^2^) is the spatial mode as shown in Fig. 3. The 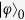 combined with the coherent state 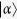 can be described as


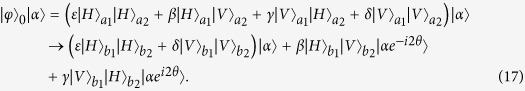


The PCM gate works as follows. From Eq. (17), if the coherent state picks up no phase shift, the state will become the even parity state 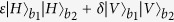. If the coherent state picks up the phase shift 2*θ*, the state will collapse to the odd parity state 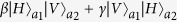. Here we should require the ±2*θ* undistinguished, which can be completed by X quadrature measurement. It can be achieved by choosing the local oscillator phase *π*/2 offset from the probe phase^17^. The error probability can be easily obtained with the same principle as described in Refs 13, 17. It can be described as 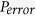 
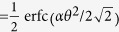
. If we choose the same coherent probe beam and weak cross-Kerr nonlinearities with *αθ*^2^ > 9, we can obtain that *P*_*error*_ is less than 10^−5^.

## Additional Information

**How to cite this article**: Sheng, Y.-B. and Zhou, L. Two-step complete polarization logic Bell-state analysis. *Sci. Rep.* **5**, 13453; doi: 10.1038/srep13453 (2015).

## Supplementary Material

Supplementary Information
