# Supplementary material for: Two-step complete polarization logic Bell-state analysis
Source: Sci Rep. 2015 Aug 26;5:13453. doi: 10.1038/srep13453 (PMC4549687; doi:10.1038/srep13453)
Supplement: Supplementary Information [file srep13453-s1.pdf]

Two-step complete polarization logic Bell-state analysis

Yu-Bo Sheng[1,2*] and Lan Zhou[2,3]
[1] *Institute of Signal Processing Transmission, Nanjing University of Posts and Telecommunications, Nanjing, 210003, China*
[2] *Key Lab of Broadband Wireless Communication and Sensor Network Technology, Nanjing University of Posts and Telecommunications, Ministry of Education, Nanjing, 210003, China*
[3] *College of Mathematics & Physics, Nanjing University of Posts and Telecommunications, Nanjing, 210003, China*
*

I. ARBITRARY C-GHZ STATE ANALYSIS

The GHZ state plays a crucial role in fundamental tests of quantum mechanics and it exhibits a conflict with local realism for non-statistical predictions of quantum mechanics [1]. One line of the research on the GHZ state analysis is based on linear optics. In 1998, Pan and Zeilinger showed that with linear optics, one can only distinguish two GHZ states among 2^N GHZ states [2]. Here N is the particle number of the GHZ state. The second line of the research on the GHZ state analysis exploits nonlinear system. In 2005, Qian *et al.* developed an efficient approach to realize the near complete GHZ state analysis [3]. In their protocol, they used the weak cross-Kerr nonlinearity to construct the PCM gate. Their protocol is also suitable for the dipole-induced transparency in a cavity-waveguide system [4]. The third line of the research uses the hyperentanglement. In 2013, Song *et al.* proposed the complete GHZ state analysis with hyperentanglement [5]. Similar to the BSA, though several protocols for GHZ states analysis were proposed, they all focus on the GHZ state encoded in the physical qubit directly. None protocol discusses the logic GHZ state analysis. From our description, it is straightforward to extend the approach of LBSA to the case of arbitrary C-GHZ state. In order to explain the C-GHZ state analysis clearly, we first let the logic qubits be $|\phi^+\rangle$ and $|\phi^-\rangle$, respectively. Therefore, the arbitrary C-GHZ state can be described as

$$\begin{aligned}
|\Phi_1^{\pm}\rangle_{N,2} &= \frac{1}{\sqrt{2}}(|\phi^+\rangle^{\otimes N} \pm |\phi^-\rangle^{\otimes N}),\\
|\Phi_2^{\pm}\rangle_{N,2} &= \frac{1}{\sqrt{2}}(|\phi^-\rangle|\phi^+\rangle^{\otimes N-1} \pm |\phi^+\rangle|\phi^-\rangle^{\otimes N-1}),\\
&\cdots,\\
|\Phi_{2^{N-1}}^{\pm}\rangle_{N,2} &= \frac{1}{\sqrt{2}}(|\phi^+\rangle^{\otimes N-1}|\phi^-\rangle \pm |\phi^-\rangle^{\otimes N-1}|\phi^+\rangle).
\end{aligned} \tag{1}$$

From Fig. 1, we first perform the Hadamard operation on each photon and make the states in Eq. (1) become

$$\begin{aligned}
|\Phi_1^{\pm}\rangle_{N,2} &= \frac{1}{\sqrt{2}}(|\phi^+\rangle^{\otimes N} \pm |\psi^+\rangle^{\otimes N}),\\
|\Phi_2^{\pm}\rangle_{N,2} &= \frac{1}{\sqrt{2}}(|\psi^+\rangle|\phi^+\rangle^{\otimes N-1} \pm |\phi^+\rangle|\psi^+\rangle^{\otimes N-1}),\\
&\cdots,\\
|\Phi_{2^{N-1}}^{\pm}\rangle_{N,2} &= \frac{1}{\sqrt{2}}(|\phi^+\rangle^{\otimes N-1}|\psi^+\rangle \pm |\psi^+\rangle^{\otimes N-1}|\phi^+\rangle).
\end{aligned} \tag{2}$$

The basic principle to distinguish the arbitrary N logic-qubit C-GHZ state can be described as follows: in the first step, according to the PCM results as shown in Fig. 1, the 2^N C-GHZ states can be divided into 2^{N-1} groups. They are $|\Phi_1^{\pm}\rangle_{N,2}, |\Phi_2^{\pm}\rangle_{N,2}, \cdots, |\Phi_{2^{N-1}}^{\pm}\rangle_{N,2}$. The second step is to distinguish the two states in each group, respectively. In order to describe this protocol clearly, we first discuss a simple case with $N = 3$. If $N = 3$, the eight C-GHZ states

* Email address: shengyb@njupt.edu.cn

can be described as

$$|\Phi_1^{\pm}\rangle_{3,2} = \frac{1}{\sqrt{2}}(|\phi^+\rangle_A|\phi^+\rangle_B|\phi^+\rangle_C \pm |\psi^+\rangle_A|\psi^+\rangle_B|\psi^+\rangle_C),$$
$$|\Phi_2^{\pm}\rangle_{3,2} = \frac{1}{\sqrt{2}}(|\psi^+\rangle_A|\phi^+\rangle_B|\phi^+\rangle_C \pm |\phi^+\rangle_A|\psi^+\rangle_B|\psi^+\rangle_C),$$
$$|\Phi_3^{\pm}\rangle_{3,2} = \frac{1}{\sqrt{2}}(|\phi^+\rangle_A|\psi^+\rangle_B|\phi^+\rangle_C \pm |\psi^+\rangle_A|\phi^+\rangle_B|\psi^+\rangle_C),$$
$$|\Phi_4^{\pm}\rangle_{3,2} = \frac{1}{\sqrt{2}}(|\phi^+\rangle_A|\phi^+\rangle_B|\psi^+\rangle_C \pm |\psi^+\rangle_A|\psi^+\rangle_B|\phi^+\rangle_C). \quad (3)$$

States $|\Phi_1^{\pm}\rangle_{3,2}$ can be described as

$$\begin{aligned}
|\Phi_1^{\pm}\rangle_{3,2} &= \frac{1}{\sqrt{2}}(|\phi^+\rangle_A|\phi^+\rangle_B|\phi^+\rangle_C \pm |\psi^+\rangle_A|\psi^+\rangle_B|\psi^+\rangle_C) = \frac{1}{\sqrt{2}}[\frac{1}{\sqrt{2}}(|H\rangle_{a_1}|H\rangle_{a_2} + |V\rangle_{a_1}|V\rangle_{a_2}) \\
&\otimes \frac{1}{\sqrt{2}}(|H\rangle_{b_1}|H\rangle_{b_2} + |V\rangle_{b_1}|V\rangle_{b_2}) \otimes \frac{1}{\sqrt{2}}(|H\rangle_{c_1}|H\rangle_{c_2} + |V\rangle_{c_1}|V\rangle_{c_2}) \pm \frac{1}{\sqrt{2}}(|H\rangle_{a_1}|V\rangle_{a_2} + |V\rangle_{a_1}|H\rangle_{a_2}) \\
&\otimes \frac{1}{\sqrt{2}}(|H\rangle_{b_1}|V\rangle_{b_2} + |V\rangle_{b_1}|H\rangle_{b_2}) \otimes \frac{1}{\sqrt{2}}(|H\rangle_{c_1}|V\rangle_{c_2} + |V\rangle_{c_1}|H\rangle_{c_2})] \\
&= \frac{1}{2\sqrt{2}}[(|H\rangle_{a_1}|H\rangle_{a_2}|H\rangle_{b_1}|H\rangle_{b_2}|H\rangle_{c_1}|H\rangle_{c_2} + |H\rangle_{a_1}|H\rangle_{a_2}|H\rangle_{b_1}|H\rangle_{b_2}|V\rangle_{c_1}|V\rangle_{c_2} \\
&+ |H\rangle_{a_1}|H\rangle_{a_2}|V\rangle_{b_1}|V\rangle_{b_2}|H\rangle_{c_1}|H\rangle_{c_2} + |V\rangle_{a_1}|V\rangle_{a_2}|H\rangle_{b_1}|H\rangle_{b_2}|H\rangle_{c_1}|H\rangle_{c_2} \\
&+ |V\rangle_{a_1}|V\rangle_{a_2}|V\rangle_{b_1}|V\rangle_{b_2}|H\rangle_{c_1}|H\rangle_{c_2} + |V\rangle_{a_1}|V\rangle_{a_2}|H\rangle_{b_1}|H\rangle_{b_2}|V\rangle_{c_1}|V\rangle_{c_2} \\
&+ |H\rangle_{a_1}|H\rangle_{a_2}|V\rangle_{b_1}|V\rangle_{b_2}|V\rangle_{c_1}|V\rangle_{c_2} + |V\rangle_{a_1}|V\rangle_{a_2}|V\rangle_{b_1}|V\rangle_{b_2}|V\rangle_{c_1}|V\rangle_{c_2}) \\
&\pm (|H\rangle_{a_1}|V\rangle_{a_2}|H\rangle_{b_1}|V\rangle_{b_2}|H\rangle_{c_1}|V\rangle_{c_2} + |H\rangle_{a_1}|V\rangle_{a_2}|H\rangle_{b_1}|V\rangle_{b_2}|V\rangle_{c_1}|H\rangle_{c_2} \\
&+ |H\rangle_{a_1}|V\rangle_{a_2}|V\rangle_{b_1}|H\rangle_{b_2}|H\rangle_{c_1}|V\rangle_{c_2} + |V\rangle_{a_1}|H\rangle_{a_2}|H\rangle_{b_1}|V\rangle_{b_2}|H\rangle_{c_1}|V\rangle_{c_2} \\
&+ |V\rangle_{a_1}|H\rangle_{a_2}|V\rangle_{b_1}|H\rangle_{b_2}|H\rangle_{c_1}|V\rangle_{c_2} + |V\rangle_{a_1}|H\rangle_{a_2}|H\rangle_{b_1}|V\rangle_{b_2}|V\rangle_{c_1}|H\rangle_{c_2} \\
&+ |H\rangle_{a_1}|V\rangle_{a_2}|V\rangle_{b_1}|H\rangle_{b_2}|V\rangle_{c_1}|H\rangle_{c_2} + |V\rangle_{a_1}|H\rangle_{a_2}|V\rangle_{b_1}|H\rangle_{b_2}|V\rangle_{c_1}|H\rangle_{c_2})],
\end{aligned} \quad (4)$$

which can be rewritten as

$$\begin{aligned}
|\Phi_1^{\pm}\rangle_{3,2} &= \frac{1}{2\sqrt{2}}[(|H\rangle_{a_1}|H\rangle_{b_1}|H\rangle_{c_1}|H\rangle_{a_2}|H\rangle_{b_2}|H\rangle_{c_2} + |H\rangle_{a_1}|H\rangle_{b_1}|V\rangle_{c_1}|H\rangle_{a_2}|H\rangle_{b_2}|V\rangle_{c_2} \\
&+ |H\rangle_{a_1}|V\rangle_{b_1}|H\rangle_{c_1}|H\rangle_{a_2}|V\rangle_{b_2}|H\rangle_{c_2} + |V\rangle_{a_1}|H\rangle_{b_1}|H\rangle_{c_1}|V\rangle_{a_2}|H\rangle_{b_2}|H\rangle_{c_2} \\
&+ |V\rangle_{a_1}|V\rangle_{b_1}|H\rangle_{c_1}|V\rangle_{a_2}|V\rangle_{b_2}|H\rangle_{c_2} + |V\rangle_{a_1}|H\rangle_{b_1}|V\rangle_{c_1}|V\rangle_{a_2}|H\rangle_{b_2}|V\rangle_{c_2} \\
&+ |H\rangle_{a_1}|V\rangle_{b_1}|V\rangle_{c_1}|H\rangle_{a_2}|V\rangle_{b_2}|V\rangle_{c_2} + |V\rangle_{a_1}|V\rangle_{b_1}|V\rangle_{c_1}|V\rangle_{a_2}|V\rangle_{b_2}|V\rangle_{c_2}) \\
&\pm (|H\rangle_{a_1}|H\rangle_{b_1}|H\rangle_{c_1}|V\rangle_{a_2}|V\rangle_{b_2}|V\rangle_{c_2} + |H\rangle_{a_1}|H\rangle_{b_1}|V\rangle_{c_1}|V\rangle_{a_2}|V\rangle_{b_2}|H\rangle_{c_2} \\
&+ |H\rangle_{a_1}|V\rangle_{b_1}|H\rangle_{c_1}|V\rangle_{a_2}|H\rangle_{b_2}|V\rangle_{c_2} + |V\rangle_{a_1}|H\rangle_{b_1}|H\rangle_{c_1}|H\rangle_{a_2}|V\rangle_{b_2}|V\rangle_{c_2} \\
&+ |V\rangle_{a_1}|V\rangle_{b_1}|H\rangle_{c_1}|H\rangle_{a_2}|H\rangle_{b_2}|V\rangle_{c_2} + |V\rangle_{a_1}|H\rangle_{b_1}|V\rangle_{c_1}|H\rangle_{a_2}|V\rangle_{b_2}|H\rangle_{c_2} \\
&+ |H\rangle_{a_1}|V\rangle_{b_1}|V\rangle_{c_1}|V\rangle_{a_2}|H\rangle_{b_2}|H\rangle_{c_2} + |V\rangle_{a_1}|V\rangle_{b_1}|V\rangle_{c_1}|H\rangle_{a_2}|H\rangle_{b_2}|H\rangle_{c_2})].
\end{aligned} \quad (5)$$

From Fig. 1, the basic principle of this protocol is to make the PCM operation between neighboring physical qubit in each logic qubit. We should point out that the PCM will not change the physical state of the photon at all, for we only require to measure the phase shift of the coherent state. Such PCM is also called the quantum nondemolition (QND) measurement. It ensures that we can perform sequential measurements on the photon. In the case of $N = 3$, we first perform the PCM operation between the photons in spatial modes a_1b_1, b_1c_1, a_2b_2 and b_2c_2 respectively. For example, in Eq. (5), item $|H\rangle_{a_1}|H\rangle_{b_1}|H\rangle_{c_1}|H\rangle_{a_2}|H\rangle_{b_2}|H\rangle_{c_2}$ will make the PCM results in spatial modes a_1b_1 and a_2b_2 both be even and the PCM results in spatial modes b_1c_1 and b_2c_2 both be even. The item $|H\rangle_{a_1}|H\rangle_{b_1}|V\rangle_{c_1}|H\rangle_{a_2}|H\rangle_{b_2}|V\rangle_{c_2}$ will make the the PCM results in spatial modes a_1b_1 and a_2b_2 both be even and the PCM results in spatial modes b_1c_1 and b_2c_2 both be odd.

In order to describe the PCM results clearly, we denote the PCM result in a_1b_1 modes as $0_{a_1b_1}$, if it is in the even parity and $1_{a_1b_1}$ if it is in the odd parity, respectively. If the initial states are $|\Phi_1^{\pm}\rangle_{3,2}$, all the possible cases of the

TABLE I: The PCM results for $|\Phi_1^{\pm}\rangle_{3,2}$, $|\Phi_2^{\pm}\rangle_{3,2}$, $|\Phi_3^{\pm}\rangle_{3,2}$ and $|\Phi_4^{\pm}\rangle_{3,2}$. Here "0" means the even parity and "1" means the odd parity, respectively.

	$\lvert\Phi_1^{\pm}\rangle_{3,2}$	$\lvert\Phi_2^{\pm}\rangle_{3,2}$	$\lvert\Phi_3^{\pm}\rangle_{3,2}$	$\lvert\Phi_4^{\pm}\rangle_{3,2}$
a_1b_1	0 0 1 1	0 0 1 1	0 0 1 1	0 0 1 1
b_1c_1	0 1 0 1	0 1 0 1	0 1 0 1	0 1 0 1
a_2b_2	0 0 1 1	1 1 0 0	1 1 0 0	0 0 1 1
b_2c_2	0 1 0 1	0 1 0 1	1 0 1 0	1 0 1 0

PCM results can be written as $0_{a_1b_1}0_{b_1c_1}0_{a_2b_2}0_{b_2c_2}$, $0_{a_1b_1}1_{b_1c_1}0_{a_2b_2}1_{b_2c_2}$, $1_{a_1b_1}0_{b_1c_1}1_{a_2b_2}0_{b_2c_2}$, or $1_{a_1b_1}1_{b_1c_1}1_{a_2b_2}1_{b_2c_2}$, respectively. All the possible PCM results for $|\Phi_1^{\pm}\rangle_{3,2}$, $|\Phi_2^{\pm}\rangle_{3,2}$, $|\Phi_3^{\pm}\rangle_{3,2}$ and $|\Phi_4^{\pm}\rangle_{3,2}$ are shown in Table I. There are 16 possible cases. From Table I, if the initial states are $|\Phi_1^{\pm}\rangle_{3,2}$, the result of PCM in the left side say a_1b_1 modes is the same as the result in the right side say a_2b_2 modes. The result of PCM in b_1c_1 modes in the left side is also the same as the PCM result in b_2c_2 modes in the right side. However, if the initial states are $|\Phi_2^{\pm}\rangle_{3,2}$, the result of PCM in the left side say a_1b_1 modes is different from the PCM result the right side a_2b_2 modes. In this way, the eight C-GHZ states can be divided into four groups, according to the PCM results. The four groups are $\{|\Phi_1^{\pm}\rangle_{3,2}\}$, $\{|\Phi_2^{\pm}\rangle_{3,2}\}$, $\{|\Phi_3^{\pm}\rangle_{3,2}\}$ and $\{|\Phi_4^{\pm}\rangle_{3,2}\}$, respectively.

The second step is to distinguish the two states in each group. We first discuss $|\Phi_1^{\pm}\rangle_{3,2}$. If the initial states are $|\Phi_1^{\pm}\rangle_{3,2}$, as shown in Table I, the PCM results are one of the results as $0_{a_1b_1}0_{b_1c_1}0_{a_2b_2}0_{b_2c_2}$, $0_{a_1b_1}1_{b_1c_1}0_{a_2b_2}1_{b_2c_2}$, $1_{a_1b_1}0_{b_1c_1}1_{a_2b_2}0_{b_2c_2}$, and $1_{a_1b_1}1_{b_1c_1}1_{a_2b_2}1_{b_2c_2}$, with the equal probability of $\frac{1}{4}$, respectively. First, if the PCM results are $0_{a_1b_1}0_{b_1c_1}0_{a_2b_2}0_{b_2c_2}$, states $|\Phi_1^{\pm}\rangle_{3,2}$ will become

$$\begin{aligned}|\Phi_1^{\pm}\rangle_{3,2} &\rightarrow \frac{1}{2}[(|H\rangle_{a_1}|H\rangle_{a_2}|H\rangle_{b_1}|H\rangle_{b_2}|H\rangle_{c_1}|H\rangle_{c_2}+|V\rangle_{a_1}|V\rangle_{a_2}|V\rangle_{b_1}|V\rangle_{b_2}|V\rangle_{c_1}|V\rangle_{c_2})\\ &\pm (|H\rangle_{a_1}|V\rangle_{a_2}|H\rangle_{b_1}|V\rangle_{b_2}|H\rangle_{c_1}|V\rangle_{c_2}+|V\rangle_{a_1}|H\rangle_{a_2}|V\rangle_{b_1}|H\rangle_{b_2}|V\rangle_{c_1}|H\rangle_{c_2})]\\ &= \frac{1}{\sqrt{2}}(|H\rangle_{a_1}|H\rangle_{b_1}|H\rangle_{c_1}\pm|V\rangle_{a_1}|V\rangle_{b_1}|V\rangle_{c_1})\otimes\frac{1}{\sqrt{2}}(|H\rangle_{a_2}|H\rangle_{b_2}|H\rangle_{c_2}\pm|V\rangle_{a_2}|V\rangle_{b_2}|V\rangle_{c_2}). \end{aligned} \tag{6}$$

Second, if the PCM results are $0_{a_1b_1}1_{b_1c_1}0_{a_2b_2}1_{b_2c_2}$, they will make $|\Phi_1^{\pm}\rangle_{3,2}$ become

$$|\Phi_1^{\pm}\rangle_{3,2} \rightarrow \frac{1}{\sqrt{2}}(|H\rangle_{a_1}|H\rangle_{b_1}|V\rangle_{c_1}\pm|V\rangle_{a_1}|V\rangle_{b_1}|H\rangle_{c_1})\otimes\frac{1}{\sqrt{2}}(|H\rangle_{a_2}|H\rangle_{b_2}|V\rangle_{c_2}\pm|V\rangle_{a_2}|V\rangle_{b_2}|H\rangle_{c_2}). \tag{7}$$

Third, if the PCM results are $1_{a_1b_1}0_{b_1c_1}1_{a_2b_2}0_{b_2c_2}$, they will make $|\Phi_1^{\pm}\rangle_{3,2}$ become

$$|\Phi_1^{\pm}\rangle_{3,2} \rightarrow \frac{1}{\sqrt{2}}(|V\rangle_{a_1}|H\rangle_{b_1}|H\rangle_{c_1}\pm|H\rangle_{a_1}|V\rangle_{b_1}|V\rangle_{c_1})\otimes\frac{1}{\sqrt{2}}(|V\rangle_{a_2}|H\rangle_{b_2}|H\rangle_{c_2}\pm|H\rangle_{a_2}|V\rangle_{b_2}|V\rangle_{c_2}). \tag{8}$$

Forth, if the PCM results are $1_{a_1b_1}1_{b_1c_1}1_{a_2b_2}1_{b_2c_2}$, they will make $|\Phi_1^{\pm}\rangle_{3,2}$ become

$$|\Phi_1^{\pm}\rangle_{3,2} \rightarrow \frac{1}{\sqrt{2}}(|H\rangle_{a_1}|V\rangle_{b_1}|H\rangle_{c_1}\pm|V\rangle_{a_1}|H\rangle_{b_1}|V\rangle_{c_1})\otimes\frac{1}{\sqrt{2}}(|H\rangle_{a_2}|V\rangle_{b_2}|H\rangle_{c_2}\pm|V\rangle_{a_2}|H\rangle_{b_2}|V\rangle_{c_2}). \tag{9}$$

The next step is only to distinguish the states $\frac{1}{\sqrt{2}}(|H\rangle_{a_1}|H\rangle_{b_1}|H\rangle_{c_1}\pm|V\rangle_{a_1}|V\rangle_{b_1}|V\rangle_{c_1})$. Certainly, if we obtain other states, such as $\frac{1}{\sqrt{2}}(|H\rangle_{a_1}|H\rangle_{b_1}|V\rangle_{c_1}\pm|V\rangle_{a_1}|V\rangle_{b_1}|H\rangle_{c_1})$ in the second case, we can perform a bit-flip operation on the c_1 photon and make them become $\frac{1}{\sqrt{2}}(|H\rangle_{a_1}|H\rangle_{b_1}|H\rangle_{c_1}\pm|V\rangle_{a_1}|V\rangle_{b_1}|V\rangle_{c_1})$.

The discrimination of the states $\frac{1}{\sqrt{2}}(|H\rangle_{a_1}|H\rangle_{b_1}|H\rangle_{c_1}\pm|V\rangle_{a_1}|V\rangle_{b_1}|V\rangle_{c_1})$ can be described as follows. As shown in Fig. 1, we first perform the Hadamard operations on each photons and make $\frac{1}{\sqrt{2}}(|H\rangle_{a_1}|H\rangle_{b_1}|H\rangle_{c_1}\pm|V\rangle_{a_1}|V\rangle_{b_1}|V\rangle_{c_1})$ become

$$\begin{aligned}\frac{1}{\sqrt{2}}(|H\rangle_{a_1}|H\rangle_{b_1}|H\rangle_{c_1}+|V\rangle_{a_1}|V\rangle_{b_1}|V\rangle_{c_1}) &\rightarrow \frac{1}{2}(|H\rangle_{a_1}|H\rangle_{b_1}|H\rangle_{c_1}+|H\rangle_{a_1}|V\rangle_{b_1}|V\rangle_{c_1}\\ &+ |V\rangle_{a_1}|H\rangle_{b_1}|V\rangle_{c_1}+|V\rangle_{a_1}|V\rangle_{b_1}|H\rangle_{c_1}),\end{aligned} \tag{10}$$

and

$$\begin{aligned}\frac{1}{\sqrt{2}}(|H\rangle_{a_1}|H\rangle_{b_1}|H\rangle_{c_1}-|V\rangle_{a_1}|V\rangle_{b_1}|V\rangle_{c_1}) &\rightarrow \frac{1}{2}(|H\rangle_{a_1}|H\rangle_{b_1}|V\rangle_{c_1}+|H\rangle_{a_1}|V\rangle_{b_1}|H\rangle_{c_1}\\ &+ |V\rangle_{a_1}|H\rangle_{b_1}|H\rangle_{c_1}+|V\rangle_{a_1}|V\rangle_{b_1}|V\rangle_{c_1}).\end{aligned} \tag{11}$$

Subsequently, we let three photons pass through three polarization beam splitters (PBSs), respectively. The PBS can transmit $|H\rangle$ polarized photon and reflect $|V\rangle$ polarized photon, respectively. Finally, by detecting the photons in each output modes, we can distinguish the state $\frac{1}{\sqrt{2}}(|H\rangle_{a_1}|H\rangle_{b_1}|H\rangle_{c_1}+|V\rangle_{a_1}|V\rangle_{b_1}|V\rangle_{c_1})$ from $\frac{1}{\sqrt{2}}(|H\rangle_{a_1}|H\rangle_{b_1}|H\rangle_{c_1}-|V\rangle_{a_1}|V\rangle_{b_1}|V\rangle_{c_1})$. If the number of $|V\rangle$ is even, it is $\frac{1}{\sqrt{2}}(|H\rangle_{a_1}|H\rangle_{b_1}|H\rangle_{c_1}+|V\rangle_{a_1}|V\rangle_{b_1}|V\rangle_{c_1})$, and the initial state is $|\Phi_1^+\rangle_{3,2}$. Otherwise, if the number of $|V\rangle$ is odd, it is $\frac{1}{\sqrt{2}}(|H\rangle_{a_1}|H\rangle_{b_1}|H\rangle_{c_1}-|V\rangle_{a_1}|V\rangle_{b_1}|V\rangle_{c_1})$, and the initial state is $|\Phi_1^-\rangle_{3,2}$.

So far, we have completely distinguished the states $|\Phi_1^\pm\rangle_{3,2}$. The other six states can be distinguished with the same principle. For example, in the first step, if the PCM results are one of $0_{a_1b_1}0_{b_1c_1}1_{a_2b_2}0_{b_2c_2}$, $0_{a_1b_1}1_{b_1c_1}1_{a_2b_2}1_{b_2c_2}$, $1_{a_1b_1}0_{b_1c_1}0_{a_2b_2}0_{b_2c_2}$, or $1_{a_1b_1}1_{b_1c_1}0_{a_2b_2}1_{b_2c_2}$, the initial states must be one of the states $|\Phi_2^\pm\rangle_{3,2}$. The second step is to distinguish $|\Phi_2^+\rangle_{3,2}$ from $|\Phi_2^-\rangle_{3,2}$. We take the PCM result $0_{a_1b_1}0_{b_1c_1}1_{a_2b_2}0_{b_2c_2}$ for example. If the PCM results are $0_{a_1b_1}0_{b_1c_1}1_{a_2b_2}0_{b_2c_2}$, $|\Phi_2^\pm\rangle_{3,2}$ becomes

$$\begin{aligned}|\Phi_2^\pm\rangle_{3,2} &\rightarrow \frac{1}{2}[(|H\rangle_{a_1}|V\rangle_{a_2}|H\rangle_{b_1}|H\rangle_{b_2}|H\rangle_{c_1}|H\rangle_{c_2}+|V\rangle_{a_1}|H\rangle_{a_2}|V\rangle_{b_1}|V\rangle_{b_2}|V\rangle_{c_1}|V\rangle_{c_2})\\ &\pm\ (|H\rangle_{a_1}|H\rangle_{a_2}|H\rangle_{b_1}|V\rangle_{b_2}|H\rangle_{c_1}|V\rangle_{c_2}+|V\rangle_{a_1}|V\rangle_{a_2}|V\rangle_{b_1}|H\rangle_{b_2}|V\rangle_{c_1}|H\rangle_{c_2})]\\ &=\ \frac{1}{\sqrt{2}}(|H\rangle_{a_1}|H\rangle_{b_1}|H\rangle_{c_1}\pm|V\rangle_{a_1}|V\rangle_{b_1}|V\rangle_{c_1})\otimes\frac{1}{\sqrt{2}}(|H\rangle_{a_2}|V\rangle_{b_2}|V\rangle_{c_2}\pm|V\rangle_{a_2}|H\rangle_{b_2}|H\rangle_{c_2}).\end{aligned}\tag{12}$$

Similarly, the second step is also to distinguish the states $\frac{1}{\sqrt{2}}(|H\rangle_{a_1}|H\rangle_{b_1}|H\rangle_{c_1}\pm|V\rangle_{a_1}|V\rangle_{b_1}|V\rangle_{c_1})$. Certainly, if the PCM results in the first step are $0_{a_1b_1}1_{b_1c_1}1_{a_2b_2}1_{b_2c_2}$, $1_{a_1b_1}0_{b_1c_1}0_{a_2b_2}0_{b_2c_2}$, or $1_{a_1b_1}1_{b_1c_1}0_{a_2b_2}1_{b_2c_2}$, they can also be distinguished with the same principle. In this way, they can be simplified to distinguish $\frac{1}{\sqrt{2}}(|H\rangle_{a_1}|H\rangle_{b_1}|H\rangle_{c_1}\pm|V\rangle_{a_1}|V\rangle_{b_1}|V\rangle_{c_1})$ after performing a bit-flip operation in the next step. Therefore, we can completely distinguish the states $|\Phi_2^\pm\rangle_{3,2}$. The other states $|\Phi_3^\pm\rangle_{3,2}$ and $|\Phi_4^\pm\rangle_{3,2}$ can also be distinguished in the same way. If the initial states are $|\Phi_3^\pm\rangle_{3,2}$, the PCM results in the first step must be one of the results $0_{a_1b_1}0_{b_1c_1}1_{a_2b_2}1_{b_2c_2}$, $0_{a_1b_1}1_{b_1c_1}1_{a_2b_2}0_{b_2c_2}$, $1_{a_1b_1}0_{b_1c_1}0_{a_2b_2}1_{b_2c_2}$, or $1_{a_1b_1}1_{b_1c_1}0_{a_2b_2}0_{b_2c_2}$, respectively. If the initial states are $|\Phi_4^\pm\rangle_{3,2}$, the PCM results in the first step must be $0_{a_1b_1}0_{b_1c_1}0_{a_2b_2}1_{b_2c_2}$, $0_{a_1b_1}1_{b_1c_1}0_{a_2b_2}0_{b_2c_2}$, $1_{a_1b_1}0_{b_1c_1}1_{a_2b_2}1_{b_2c_2}$, or $1_{a_1b_1}1_{b_1c_1}1_{a_2b_2}0_{b_2c_2}$, respectively. Therefore, in the second step, we only need to distinguish the states $\frac{1}{\sqrt{2}}(|H\rangle_{a_1}|H\rangle_{b_1}|H\rangle_{c_1}\pm|V\rangle_{a_1}|V\rangle_{b_1}|V\rangle_{c_1})$ in each group. In this way, all eight states $|\Phi_1^\pm\rangle_{3,2}$, $|\Phi_2^\pm\rangle_{3,2}$, $|\Phi_3^\pm\rangle_{3,2}$ and $|\Phi_4^\pm\rangle_{3,2}$ can be completely distinguished.

It is straightforward to extend this protocol to distinguish the C-GHZ state with N logic qubits as shown in Eq. (2). The basic principle is also shown in Fig. 1. In the first step, we perform the PCM operations on the photons in spatial modes a_1b_1, b_1c_1, $\cdots$, m_1n_1 in the left side and a_2b_2, b_2c_2, $\cdots$, m_2n_2 in the right side. In each side, we should perform $N-1$ PCM operations. Interestingly, if the initial states are $|\Phi_1^\pm\rangle_{N,2}$, the PCM result in the left side always equals to the result in the right side in the correspond position. It means that the PCM result in a_1b_1 modes equals to that in a_2b_2 modes. The PCM result in b_1c_1 modes equals to that in b_2c_2 modes, $\cdots$, and the PCM result in m_1n_1 modes equals to that in m_2n_2 modes. The PCM results in the left side say $P_{a_1b_1}P_{b_1c_1}\cdots P_{m_1n_1}(P=0,1)$ have 2^{N-1} possible cases with the same probability $\frac{1}{2^{N-1}}$. They are $0_{a_1b_1}0_{b_1c_1}\cdots 0_{m_1n_1}$, $0_{a_1b_1}0_{b_1c_1}\cdots 1_{m_1n_1}$, $\cdots$, $1_{a_1b_1}1_{b_1c_1}\cdots 1_{m_1n_1}$. Therefore, the PCM results in the left side combined with the right side must be $0_{a_1b_1}0_{b_1c_1}\cdots 0_{m_1n_1}0_{a_2b_2}0_{b_2c_2}\cdots 0_{m_2n_2}$, $0_{a_1b_1}0_{b_1c_1}\cdots 1_{m_1n_1}0_{a_2b_2}0_{b_2c_2}\cdots 1_{m_2n_2}$, $\cdots$, or $1_{a_1b_1}1_{b_1c_1}\cdots 1_{m_1n_1}1_{a_2b_2}1_{b_2c_2}\cdots 1_{m_2n_2}$. For example, if the PCM results are $0_{a_1b_1}0_{b_1c_1}\cdots 0_{m_1n_1}0_{a_2b_2}0_{b_2c_2}\cdots 0_{m_2n_2}$, the states $|\Phi_1^\pm\rangle_{N,2}$ will collapse to

$$\begin{aligned}|\Phi_1^\pm\rangle_{N,2} &\rightarrow \frac{1}{2}[(|H\rangle_{a_1}|H\rangle_{a_2}|H\rangle_{b_1}|H\rangle_{b_2}\cdots|H\rangle_{n_1}|H\rangle_{n_2}+|V\rangle_{a_1}|V\rangle_{a_2}|V\rangle_{b_1}|V\rangle_{b_2}\cdots|V\rangle_{n_1}|V\rangle_{n_2})\\ &\pm\ (|H\rangle_{a_1}|V\rangle_{a_2}|H\rangle_{b_1}|V\rangle_{b_2}\cdots|H\rangle_{n_1}|V\rangle_{n_2}+|V\rangle_{a_1}|H\rangle_{a_2}|V\rangle_{b_1}|H\rangle_{b_2}\cdots|V\rangle_{n_1}|H\rangle_{n_2})]\\ &=\ \frac{1}{\sqrt{2}}(|H\rangle_{a_1}|H\rangle_{b_1}\cdots|H\rangle_{n_1}\pm|V\rangle_{a_1}|V\rangle_{b_1}\cdots|V\rangle_{n_1})\otimes\frac{1}{\sqrt{2}}(|H\rangle_{a_2}|H\rangle_{b_2}\cdots|H\rangle_{n_2}\pm|V\rangle_{a_2}|V\rangle_{b_2}\cdots|V\rangle_{n_2}).\end{aligned}\tag{13}$$

The second step is to distinguish $\frac{1}{\sqrt{2}}(|H\rangle_{a_1}|H\rangle_{b_1}\cdots|H\rangle_{n_1}+|V\rangle_{a_1}|V\rangle_{b_1}\cdots|V\rangle_{n_1})$ from $\frac{1}{\sqrt{2}}(|H\rangle_{a_1}|H\rangle_{b_1}\cdots|H\rangle_{n_1}-|V\rangle_{a_1}|V\rangle_{b_1}\cdots|V\rangle_{n_1})$. As shown in Fig. 1, after performing the Hadamard operation on each photons, we can make

$$\begin{aligned}&\frac{1}{\sqrt{2}}(|H\rangle_{a_1}|H\rangle_{b_1}\cdots|H\rangle_{n_1}+|V\rangle_{a_1}|V\rangle_{b_1}\cdots|V\rangle_{n_1})\rightarrow(\frac{1}{\sqrt{2}})^{N+1}[(|H\rangle_{a_1}+|V\rangle_{a_1})(|H\rangle_{b_1}+|V\rangle_{b_1})\cdots(|H\rangle_{n_1}+|V\rangle_{n_1})\\ &+(|H\rangle_{a_1}-|V\rangle_{a_1})(|H\rangle_{b_1}-|V\rangle_{b_1})\cdots(|H\rangle_{n_1}-|V\rangle_{n_1})],\end{aligned}\tag{14}$$

and

$$\begin{aligned}&\frac{1}{\sqrt{2}}(|H\rangle_{a_1}|H\rangle_{b_1}\cdots|H\rangle_{n_1}-|V\rangle_{a_1}|V\rangle_{b_1}\cdots|V\rangle_{n_1})\rightarrow(\frac{1}{\sqrt{2}})^{N+1}[(|H\rangle_{a_1}+|V\rangle_{a_1})(|H\rangle_{b_1}+|V\rangle_{b_1})\cdots(|H\rangle_{n_1}+|V\rangle_{n_1})\\ &-(|H\rangle_{a_1}-|V\rangle_{a_1})(|H\rangle_{b_1}-|V\rangle_{b_1})\cdots(|H\rangle_{n_1}-|V\rangle_{n_1})].\end{aligned}\tag{15}$$

After passing through the PBSs, if the number of $|V\rangle$ is even, it must be $\frac{1}{\sqrt{2}}(|H\rangle_{a_1}|H\rangle_{b_1}\cdots|H\rangle_{n_1} + |V\rangle_{a_1}|V\rangle_{b_1}\cdots|V\rangle_{n_1})$, and the initial state is $|\Phi_1^+\rangle_{N,2}$. If the number of $|V\rangle$ is odd, it must be $\frac{1}{\sqrt{2}}(|H\rangle_{a_1}|H\rangle_{b_1}\cdots|H\rangle_{n_1} - |V\rangle_{a_1}|V\rangle_{b_1}\cdots|V\rangle_{n_1})$, and the initial state is $|\Phi_1^-\rangle_{N,2}$. Certainly, if the PCM results are $1_{a_1b_1}0_{b_1c_1}\cdots0_{m_1n_1}1_{a_2b_2}0_{b_2c_2}\cdots0_{m_2n_2}$ in first step, the states $|\Phi_1^\pm\rangle_{N,2}$ will collapse to

$$|\Phi_1^\pm\rangle_{N,2} \rightarrow \frac{1}{\sqrt{2}}(|V\rangle_{a_1}|H\rangle_{b_1}\cdots|H\rangle_{n_1} \pm |H\rangle_{a_1}|V\rangle_{b_1}\cdots|V\rangle_{n_1}) \otimes \frac{1}{\sqrt{2}}(|V\rangle_{a_2}|H\rangle_{b_2}\cdots|H\rangle_{n_2} \pm |H\rangle_{a_2}|V\rangle_{b_2}\cdots|V\rangle_{n_2}). \quad (16)$$

After performing a bit-flip operation on the photon in spatial mode a_1, which will make $\frac{1}{\sqrt{2}}(|V\rangle_{a_1}|H\rangle_{b_1}\cdots|H\rangle_{n_1} \pm |H\rangle_{a_1}|V\rangle_{b_1}\cdots|V\rangle_{n_1})$ become $\frac{1}{\sqrt{2}}(|H\rangle_{a_1}|H\rangle_{b_1}\cdots|H\rangle_{n_1} \pm |V\rangle_{a_1}|V\rangle_{b_1}\cdots|V\rangle_{n_1})$, it can be distinguished with the same method described above. All 2^{N-1} cases can be distinguished with the same principle.

Interestingly, if the initial states are $|\Phi_2^\pm\rangle_{N,2}$, the PCM result in a_1b_1 is always different from the PCM result in a_2b_2. The other PCM results in the left side equal to that in the right side. The PCM results in the left side have 2^{N-1} possible cases. The total PCM results can be written as $0_{a_1b_1}0_{b_1c_1}\cdots0_{m_1n_1}1_{a_2b_2}0_{b_2c_2}\cdots0_{m_2n_2}$, $0_{a_1b_1}0_{b_1c_1}\cdots1_{m_1n_1}1_{a_2b_2}0_{b_2c_2}\cdots1_{m_2n_2}$, $\cdots$, or $1_{a_1b_1}1_{b_1c_1}\cdots1_{m_1n_1}0_{a_2b_2}1_{b_2c_2}\cdots1_{m_2n_2}$. If the PCM results are $0_{a_1b_1}0_{b_1c_1}\cdots0_{m_1n_1}1_{a_2b_2}0_{b_2c_2}\cdots0_{m_2n_2}$, the states $|\Phi_2^\pm\rangle_{N,2}$ will project to

$$|\Phi_2^\pm\rangle_{N,2} \rightarrow \frac{1}{\sqrt{2}}(|H\rangle_{a_1}|H\rangle_{b_1}\cdots|H\rangle_{n_1} \pm |V\rangle_{a_1}|V\rangle_{b_1}\cdots|V\rangle_{n_1}) \otimes \frac{1}{\sqrt{2}}(|V\rangle_{a_2}|H\rangle_{b_2}\cdots|H\rangle_{n_2} \pm |H\rangle_{a_2}|V\rangle_{b_2}\cdots|V\rangle_{n_2}). \quad (17)$$

The second step is also to distinguish the states $\frac{1}{\sqrt{2}}(|H\rangle_{a_1}|H\rangle_{b_1}\cdots|H\rangle_{n_1} \pm |V\rangle_{a_1}|V\rangle_{b_1}\cdots|V\rangle_{n_1})$, which has been described in the former.

If the initial states are $|\Phi_3^\pm\rangle_{N,2}$, we can find that both the PCM results in a_1b_1 and b_1c_1 are different from a_2b_2 and b_2c_2, respectively, while the other PCM results in the left side equal to that in the right side. For example, if the PCM results are $0_{a_1b_1}0_{b_1c_1}0_{c_1d_1}\cdots0_{m_1n_1}1_{a_2b_2}1_{b_2c_2}0_{c_2d_2}\cdots0_{m_2n_2}$, states $|\Phi_3^\pm\rangle_{N,2}$ will project to

$$|\Phi_3^\pm\rangle_{N,2} \rightarrow \frac{1}{\sqrt{2}}(|H\rangle_{a_1}|H\rangle_{b_1}\cdots|H\rangle_{n_1} \pm |V\rangle_{a_1}|V\rangle_{b_1}\cdots|V\rangle_{n_1}) \otimes \frac{1}{\sqrt{2}}(|H\rangle_{a_2}|V\rangle_{b_2}|H\rangle_{c_2}\cdots|H\rangle_{n_2} \pm |V\rangle_{a_2}|H\rangle_{b_2}|V\rangle_{c_2}\cdots|V\rangle_{n_2}). \quad (18)$$

The next step is also to distinguish the states $\frac{1}{\sqrt{2}}(|H\rangle_{a_1}|H\rangle_{b_1}\cdots|H\rangle_{n_1} \pm |V\rangle_{a_1}|V\rangle_{b_1}\cdots|V\rangle_{n_1})$. If it is $\frac{1}{\sqrt{2}}(|H\rangle_{a_1}|H\rangle_{b_1}\cdots|H\rangle_{n_1} + |V\rangle_{a_1}|V\rangle_{b_1}\cdots|V\rangle_{n_1})$, the initial state must be $|\Phi_3^+\rangle_{N,2}$. Otherwise, it must be $|\Phi_3^-\rangle_{N,2}$. If the initial state is the arbitrary stats $|\Phi_k^\pm\rangle_{N,2}$ ($k = 1, 2, \cdots 2^{N-1}$), it can be distinguished in the same way. In this way, we can completely distinguish the C-GHZ state as shown in Eq. (2).

We showed that the arbitrary C-GHZ state can also be completely distinguished in the same way. As shown in Fig. 1, in the first step, both the left side and right side perform $N-1$ PCM operations. According to the PCM operations, we can judge that the initial states must be one of the following groups, say $\{|\Phi_1^\pm\rangle_{N,2}\}$, $\{|\Phi_2^\pm\rangle_{N,2}\}$, $\cdots$, $\{|\Phi_{2^{N-1}}^\pm\rangle_{N,2}\}$. In the second step, we are only required to distinguish the conventional polarized GHZ states $\frac{1}{\sqrt{2}}(|H\rangle_{a_1}|H\rangle_{b_1}\cdots|H\rangle_{n_1} \pm |V\rangle_{a_1}|V\rangle_{b_1}\cdots|V\rangle_{n_1})$. After performing the Hadamard operations, they can be completely distinguished according to the number of $|V\rangle$. If the number of $|V\rangle$ is even, it must be $\frac{1}{\sqrt{2}}(|H\rangle_{a_1}|H\rangle_{b_1}\cdots|H\rangle_{n_1} + |V\rangle_{a_1}|V\rangle_{b_1}\cdots|V\rangle_{n_1})$. Otherwise, it must be $\frac{1}{\sqrt{2}}(|H\rangle_{a_1}|H\rangle_{b_1}\cdots|H\rangle_{n_1} - |V\rangle_{a_1}|V\rangle_{b_1}\cdots|V\rangle_{n_1})$. In this way, all the C-GHZ states can be completely distinguished.

In our protocol, the logic qubit is encoded in the polarized Bell states $|\phi^+\rangle$ and $|\phi^-\rangle$. Actually, the logic qubit in generalized concatenated GHZ state can be the M-particle GHZ state. Therefore, the generalized logic Bell states can be written as

$$\begin{aligned}
|\Phi_M^\pm\rangle_{AB} &= \frac{1}{\sqrt{2}}(|GHZ_M^+\rangle_A|GHZ_M^+\rangle_B \pm |GHZ_M^-\rangle_A|GHZ_M^-\rangle_B), \\
|\Psi_M^\pm\rangle_{AB} &= \frac{1}{\sqrt{2}}(|GHZ_M^+\rangle_A|GHZ_M^-\rangle_B \pm |GHZ_M^-\rangle_A|GHZ_M^+\rangle_B). \quad (19)
\end{aligned}$$

The generalized C-GHZ state can be written as

$$\begin{aligned}
|\Phi_1^{\pm}\rangle_{N,M} &= \frac{1}{\sqrt{2}}(|GHZ_M^{+}\rangle^{\otimes N} \pm |GHZ_M^{-}\rangle^{\otimes N}),\\
|\Phi_2^{\pm}\rangle_{N,M} &= \frac{1}{\sqrt{2}}(|GHZ_M^{-}\rangle|GHZ_M^{+}\rangle^{\otimes N-1} \pm |GHZ_M^{+}\rangle|GHZ_M^{-}\rangle^{\otimes N-1}),\\
&\cdots\\
|\Phi_{2^{N-1}}^{\pm}\rangle_{N,M} &= \frac{1}{\sqrt{2}}(|GHZ_M^{+}\rangle^{\otimes N-1}|GHZ_M^{-}\rangle \pm |GHZ_M^{-}\rangle^{\otimes N-1}|GHZ_M^{+}\rangle).
\end{aligned} \tag{20}$$

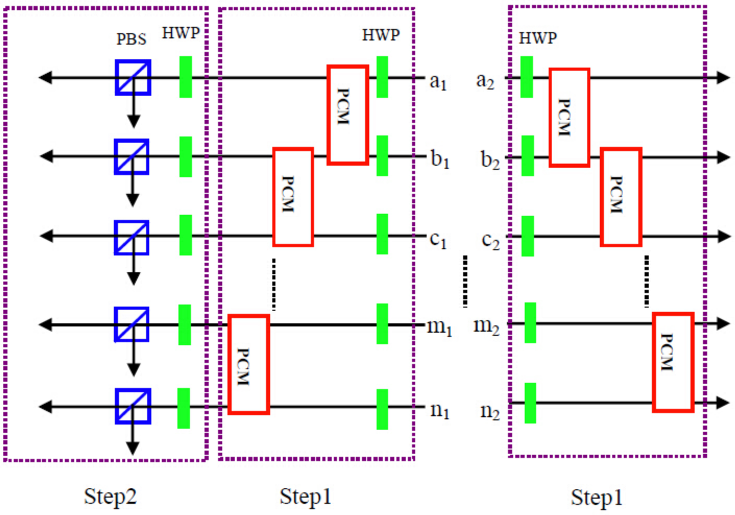

FIG. 1: A schematic drawing of distinguishing the C-GHZ state.

Interestingly, the logic Bell states and the C-GHZ states shown in Eqs. (19) and (20) can also be completely distinguished with the same method. The increased photon number in each logic qubit does not affect the distinguishing of the logic qubit entanglement. Because in C-GHZ state analysis, the number of C-GHZ state is only decided by the logic qubit number N. In order to distinguish the logic Bell states and the C-GHZ states described in Eqs. (19) and (20), we first transform the $|GHZ_M^{\pm}\rangle$ to $|\phi^{\pm}\rangle$, respectively. We take $M = 3$ for example. If $M = 3$, Eq.(20) can be rewritten as

$$\begin{aligned}
|\Phi_1^{\pm}\rangle_{N,3} &= \frac{1}{\sqrt{2}}[(\frac{1}{\sqrt{2}}(|HHH\rangle + |VVV\rangle))^{\otimes N} \pm (\frac{1}{\sqrt{2}}(|HHH\rangle - |VVV\rangle))^{\otimes N}],\\
|\Phi_2^{\pm}\rangle_{N,3} &= \frac{1}{\sqrt{2}}[\frac{1}{\sqrt{2}}(|HHH\rangle - |VVV\rangle)(\frac{1}{\sqrt{2}}(|HHH\rangle + |VVV\rangle))^{\otimes N-1}\\
&\pm \frac{1}{\sqrt{2}}(|HHH\rangle + |VVV\rangle)(\frac{1}{\sqrt{2}}(|HHH\rangle - |VVV\rangle))^{\otimes N-1}],\\
&\cdots,\\
|\Phi_{2^{N-1}}^{\pm}\rangle_{N,3} &= \frac{1}{\sqrt{2}}[(\frac{1}{\sqrt{2}}(|HHH\rangle + |VVV\rangle))^{\otimes N-1}\frac{1}{\sqrt{2}}(|HHH\rangle - |VVV\rangle)\\
&\pm (\frac{1}{\sqrt{2}}(|HHH\rangle - |VVV\rangle))^{\otimes N-1}\frac{1}{\sqrt{2}}(|HHH\rangle + |VVV\rangle)].
\end{aligned} \tag{21}$$

We perform the Hadmard operation on the third photon in each logic qubit, which will make

$$\begin{aligned}
|\Phi_1^{\pm}\rangle_{N,3} &= \frac{1}{\sqrt{2}}[(\frac{1}{\sqrt{2}}(|HHH\rangle + |VVV\rangle))^{\otimes N} \pm (\frac{1}{\sqrt{2}}(|HHH\rangle - |VVV\rangle))^{\otimes N}]\\
&\rightarrow \frac{1}{\sqrt{2}}[(\frac{1}{\sqrt{2}}(|HH\rangle\frac{1}{\sqrt{2}}(|H\rangle + |V\rangle) + |VV\rangle\frac{1}{\sqrt{2}}(|H\rangle - |V\rangle)))^{\otimes N}\\
&\pm (\frac{1}{\sqrt{2}}(|HH\rangle\frac{1}{\sqrt{2}}(|H\rangle + |V\rangle) - |VV\rangle\frac{1}{\sqrt{2}}(|H\rangle - |V\rangle)))^{\otimes N}]
\end{aligned}$$

$$= \frac{1}{\sqrt{2}}[(|\phi^+\rangle|H\rangle + |\phi^-\rangle|V\rangle)^{\otimes N} \pm (|\phi^-\rangle|H\rangle + |\phi^+\rangle|V\rangle)^{\otimes N}]. \quad (22)$$

From above description, we measure the photon in each logic qubit, in the basis $\{|H\rangle, |V\rangle\}$. If the measurement result is $|H\rangle$, the remained two-photon state in each logic qubit is $|\phi^+\rangle$. On the other hand, if the measurement result is $|V\rangle$, the remained two-photon state is $|\phi^-\rangle$. In this way, we perform a phase flip operation to convert it to $|\phi^+\rangle$. If all the measurement results are $|H\rangle$, states $|\Phi_1^{\pm}\rangle_{N,3}$ can be completely converted to $|\Phi_1^{\pm}\rangle_{N,2}$. With the same principle, $|\Phi_2^{\pm}\rangle_{N,3}$ can be converted to $|\Phi_2^{\pm}\rangle_{N,2}$, and $|\Phi_{2^{N-1}}^{\pm}\rangle_{N,3}$ can be converted to $|\Phi_{2^{N-1}}^{\pm}\rangle_{N,2}$, respectively. Certainly, if $M > 3$, in each logic qubit, we should perform $M-2$ Hadamard operation on each $M-2$ photons and finally measure these $M-2$ photons. In each logic qubit, by measuring $M-2$ photons in each logic qubit, the states $|GHZ_M^+\rangle$ will become $|\phi^+\rangle$, and $|GHZ_M^-\rangle$ will become $|\phi^-\rangle$, if the number of $|V\rangle$ is even. Otherwise, $|GHZ_M^+\rangle$ will become $|\phi^-\rangle$, and $|GHZ_M^-\rangle$ will become $|\phi^+\rangle$. In this way, we can convert $|\Phi_1^{\pm}\rangle_{N,M}$ to $|\Phi_1^{\pm}\rangle_{N,2}$, $|\Phi_2^{\pm}\rangle_{N,M}$ to $|\Phi_2^{\pm}\rangle_{N,2}$, $\cdots$, and $|\Phi_{2^{N-1}}^{\pm}\rangle_{N,2}$ to $|\Phi_{2^{N-1}}^{\pm}\rangle_{N,M}$, respectively. In the next step, with the same principle, we can perform the LBSA and C-GHZ analysis completely.

[1] Pan, J.-W. et al. Multiphoton entanglement and interferometry. *Rev. Mod. Phys.* **84**, 777-838 (2012).

[2] Pan, J.-W. & Zeilinger, A. Greenberger-Horne-Zeilinger-state analyzer. *Phys. Rev. A* **57**, 2208-2211 (1998).

[3] Qian, J., Feng, X.-L. & Gong, S.-Q. Universal Greenberger-Horne-Zeilinger-state analyzer based on two-photon polarization parity detection. *Phys. Rev. A* **72**, 052308 (2005).

[4] Qian, J., Qian, Y., Feng, X. L., Yang, T. & Gong, S. Q. Generation and discrimination of Greenberger-Horne-Zeilinger states using dipole-induced transparency in a cavity-waveguide system. *Phys. Rev. A* **75**, 032309 (2007).

[5] Song, S. Y., Cao, Y., Sheng, Y. B. & Long, G. L. Complete Greenberger-Horne-Zeilinger state analyzer using hyperentanglement. Quant. Inf. Precess. **12**, 381-393 (2013).
